# Effect of Electrical Stimulation on Diabetic Human Skin Fibroblast Growth and the Secretion of Cytokines and Growth Factors Involved in Wound Healing

**DOI:** 10.3390/biology10070641

**Published:** 2021-07-09

**Authors:** Atieh Abedin-Do, Ze Zhang, Yvan Douville, Mireille Méthot, Mahmoud Rouabhia

**Affiliations:** 1 Groupe de Recherche en Écologie Buccale, Faculté de Médecine Dentaire, Université Laval, Québec, QC G1V 0A6, Canada; atieh-sadat.abedin-do.1@ulaval.ca; 2 Axe Médecine Régénératrice, Centre de Recherche du CHU de Québec, Département de Chirurgie, Faculté de Médecine, Université Laval, Québec, QC G1V 0A6, Canada; Ze.zhang@fmed.ulaval.ca (Z.Z.); Yvan.douville@fmed.ulaval.ca (Y.D.); mireillemethot@hotmail.com (M.M.)

**Keywords:** diabetics, foot ulcers, fibroblasts, electrical stimulation, wound healing, cytokines, growth factors

## Abstract

**Simple Summary:**

With the number of diabetic patients on the rise, diabetes has become a major health issue affecting millions of people worldwide. One complication of diabetes is foot ulcers, which are difficult to repair and are thus associated with major clinical problems that may lead to foot amputation and even patient death. The delayed repair of diabetic foot ulcers is due to the slow growth of one of the cell types involved in wound healing, namely, fibroblasts. Fibroblasts inhabit deep skin tissue. Post-wound, they grow and produce skin tissues to enable other cells to close the wound. Even though normal fibroblast growth can be increased by electrical stimulation, it is not clear whether diabetic fibroblast also responds to electrical stimulation. We demonstrated for the first time that a weak direct current electrical field increased diabetic fibroblast growth. The use of electrical stimulation could thus potentially help heal diabetic foot ulcers and ultimately improve patient health and well-being.

**Abstract:**

Diabetic foot ulcers are indicative of an impaired wound healing process. This delay may be resolved through electrical stimulation (ES). The goal of the present study was to evaluate the effect of ES on diabetic fibroblast adhesion and growth, and the secretion of cytokines and growth factors. Diabetic human skin fibroblasts (DHSF) were exposed to various intensities of direct current ES (100, 80, 40 and 20 mV/mm). The effect of ES on fibroblast adhesion and growth was evaluated using Hoechst staining, MTT and trypan blue exclusion assays. The secretion of cytokine and growth factor was assessed by cytokine array and ELISA assay. The long-term effects of ES on DHSF shape and growth were determined by optical microscopy and cell count. We demonstrated that ES at 20 and 40 mV/mm promoted cell adhesion, viability and growth. ES also decreased the secretion of pro-inflammatory cytokines IL-6 and IL-8 yet promoted growth factor FGF7 secretion during 48 h post-ES. Finally, the beneficial effect of ES on fibroblast growth was maintained up to 5 days post-ES. Overall results suggest the possible use of low-intensity direct current ES to promote wound healing in diabetic patients.

## 1. Introduction

Wound repair occurs when the skin is damaged [1,2]. The wound healing process includes four phases: hemostasis, inflammation, proliferation and remodeling [3,4]. These phases involve different cell types such as fibroblasts and keratinocytes [5,6] and multiple mediators, including growth factors and cytokines [7]. These cytokines and growth factors are important in all phases of the wound healing process.

Diabetes is a major health issue affecting millions of people worldwide, and its prevalence is increasing [8,9]. The World Health Organization (WHO) estimated that by the year 2030, diabetes would become the seventh leading cause of death [10]. Diabetics have a declined ability to metabolize glucose, leading to hyperglycemia, which can compromise wound healing processes and cause frequent and chronic leg or foot ulcers, particularly when poor circulation of the lower extremities is a concomitant issue of diabetic patients [11]. Diabetic ulcers are associated with major clinical problems that may lead to severe outcomes, including deterioration of patient health and increased amputation and mortality rates [12].

At the cellular level, diabetic ulcers display increased inflammatory cells, abnormal synthesis of connective tissues and inadequate tissue epithelialization [13]. This sharply contrasts the different phases of normal healing in a healthy individual. These phases include an inflammatory phase, in which neutrophils and macrophages clean up debris and pathogens along with growth factors and other cytokines and cells, followed by a cell proliferation phase (which overlaps the inflammatory phase) during which new tissue, new blood vessels (angiogenesis) and matrix construction are initiated to fill and repair the wounded area. The wound healing process ends with a remodeling phase to increase the tensile strength of the extracellular matrix and reduce the blood supply to the wounded area [14]. Even though the wound healing process involves different cell types, fibroblasts are the primary cells involved in tissue reconstruction [15]. 

It has been reported that fibroblasts from chronic non-healing wounds (such as diabetic ulcers) display abnormal phenotypes, such as decreased proliferation, altered patterns of cytokine release, early senescence [16] and abnormal metalloproteinase activity [17]. To help these dysfunctional fibroblasts in diabetic patients to become more active in wound healing, allogeneic fibroblasts were tested by being grafted through a bi-layered skin substitute. These grafted allogeneic fibroblasts did not persist indefinitely yet did serve as a source of growth factors and cytokines to support the function of the patient’s own cells [18].

Stimulating the diabetic patient’s own fibroblasts can also be achieved through electrical stimulation (ES). Research with fibroblasts extracted from non-diabetic donors has shown that conductive polymer-based ES could not only promote the proliferation of fibroblasts but also increase the capacity of these cells to produce extracellular matrix (ECM). Furthermore, fibroblasts exposed to ES have also been shown to secrete higher levels of cytokines and growth factors, compared to non-exposed cells [19,20].

We recently demonstrated that ES upregulated α-SMA expression in normal fibroblasts [21]. The positive effects of ES on fibroblasts extracted from healthy donors could thus apply to fibroblasts from diabetic patients. The goals of the present study were to extract and culture diabetic human fibroblasts and evaluate the effects of ES on their proliferation and cytokine secretion. The long-term effects of ES on diabetic human fibroblast behaviors were also assessed.

## 2. Materials and Methods

### 2.1. Preparation of Conductive PPy/HE/PLLA Membranes

Polypyrrole (PPy) particles were formed in a water-in-oil emulsion containing pyrrole, FeCl_3_, H_2_O_2_ and heparin (HE) [19,21]. This emulsion was maintained under vigorous stirring overnight at room temperature. The PPy/HE particles were collected, washed with methanol to remove the emulsifier and impurities, and mixed thereafter with a PLLA solution in chloroform at a 1:9 weight ratio (PPy:PLLA). The mixture was transferred into a glass petri dish and kept at room temperature (RT) in a fume hood to evaporate the chloroform, which led to the formation of the PPy/HE/PLLA membranes. The membranes were then washed 5 × 2 h with deionized water and dried overnight at RT. The conductivity of the PPy/HE/PLLA membranes was assessed with a four-point probe, while the thickness was measured with a digital thickness gauge model MTG-DX2 (Rex Gauge, Buffalo Grove, IL, USA). The membranes were then cut into the appropriate size, mounted onto a homemade electrical tissue culture device and sterilized with 75% ethanol, followed by extensive washes with sterile PBS and culture medium prior to cell culture. The PPy/HE/PLLA membranes in the device were assessed for conductivity using a digital multi-meter (Keithley, Cleveland, OH, USA).

### 2.2. Primary Diabetic Human Skin Fibroblast Culture

Diabetic human skin fibroblasts (DHSF) were isolated from skin tissues collected from diabetic patients (61 to 80 years old) following leg amputation surgery. Skin samples were collected following each patient’s informed consent (Research Project 2014-1733, B13-07-1733) and the approval of the Université Laval-CHU Ethics Committee. Diabetic skin fibroblasts were extracted from the dermis following treatment with 0.125 U/mL of collagenase-P (Boehringer Mannheim, Laval, QC, Canada) for 90 min at 37 °C. The extracted fibroblasts were then seeded in 75-cm^2^ culture flasks and grown in Dulbecco’s modified Eagle’s medium (DMEM) supplemented with 10% fetal bovine serum in a humidified incubator at 37 °C with 5% CO_2_. The fibroblasts were then subcultured and used at passages 4 to 6.

### 2.3. Biocompatibility of the Conductive Membranes

To ensure that the PPy/HE/PLLA membranes did not overheat when connected to the current, they were assembled in the electrical cell culture device, where each well was filled with 3 mL of culture medium, and were incubated thereafter in a 5% CO_2_ incubator at 37 °C. The membranes were then supplied with a potential gradient of 100 mV/mm for 24 h. The temperature of the culture medium on top of each membrane was measured every 2 h with a VWR^®^ Dual Channel Thermometer (VWR International, Mississauga, ON, Canada). In another set of experiments, we determined the PPy/HE/PLLA membrane cytocompatibility with the DHSF. Briefly, using the same electrical cell culture device, DHSF (5 × 10^4^ cells/cm^2^) were seeded and cultured for 48 and 96 h on the PPy/HE/PLLA membranes. The cells were then fixed with a 4% paraformaldehyde solution and stained with Hoechst 33342 (Sigma-Aldrich, Oakville, ON, Canada). The stained cells were observed under an epifluorescence microscope and photographed.

### 2.4. Safe ES Intensities for the DHSF

**Figure 1:**
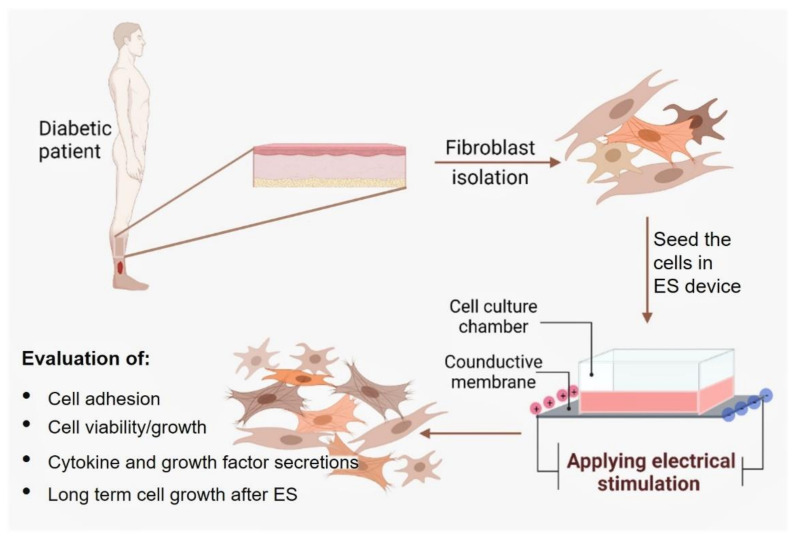
Diagram of the experimental protocol.

DHSF were seeded (5 × 10^4^ cells/cm^2^) on the membranes in the electrical cell culture device and cultured for 24 h at 37 °C in a 5% CO_2_ humid atmosphere. ES at various intensities (100, 80, 60, 40 and 20 mV/mm) were then applied or not to the cells for 6 or 24 h. Following each stimulation period, the culture medium was refreshed and the cells were cultured for an additional 48 h. The selected ES intensities and exposures times were chosen based on previously published research using normal human skin fibroblasts [19,21]. At the end of the experiment, cell viability and cytotoxicity were ascertained by MTT and LDH assays, respectively (Figure 1).

### 2.5. MTT Assay

The metabolic activity of the fibroblast cultures was used to evaluate cell growth and was measured by the 3-(4,5-dimethylthiazol-2-yl)-2,5-diphenyltetrazolium bromide (MTT) assay, as we previously reported [22]. Each experimental condition was repeated in quadruplicate.

### 2.6. LDH Activity 

Following ES, culture supernatants were collected either immediately or after an additional post-ES culture of 48 h and were subsequently subjected to an LDH cytotoxicity assay (Promega, Madison, WI, USA). Briefly, 50 μL of each supernatant was transferred to the wells of a 96-well flat-bottom plate along with 50 μL of the reconstituted substrate mix, and incubated thereafter in the dark at RT for 30 min. The absorbance of each well was read at 490 nm using an X-Mark microplate spectrophotometer (Bio-Rad, Mississauga, ON, Canada).

### 2.7. Cell Adhesion Following ES 

DHSF were seeded onto PPy/HE/PLLA membranes and immediately subjected to ES at either 20 or 40 mV/mm for 6 and 24 h. Immediately following the ES regime, the cells were fixed with a 4% paraformaldehyde solution and stained with Hoechst 33342 (Sigma-Aldrich, Oakville, ON, Canada). The stained cells were observed under an epifluorescence microscope and photographed.

### 2.8. Fibroblast Growth after Exposure to the Safe ES Regime 

DHSF were cultured on the membranes for 24 h and exposed or not to 20 or 40 mV/mm for 6 and 24 h (the safe ES regimes identified in previous experiment). Following ES, the cells were cultured for 48 h, then detached from the membranes by means of a 0.05%-trypsin-0.01 EDTA solution, washed twice with culture medium and subjected to a trypan blue exclusion assay. Briefly, 20 μL of each cell suspension was mixed with 20 μL of 0.4% trypan blue solution. The non-stained cells (viable cells) were counted using a hemocytometer. The cell count was performed in quadruplicate for each condition. 

### 2.9. Cytokine Array and ELISA Assay

Immediately following ES, culture supernatants from the DHSF cultures exposed or not to 20 or 40 mV/mm for 6 and 24 h were collected, with the culture medium refreshed for each well. The cells were cultured again and the supernatants were collected 48 h post-exposure to ES for a cytokine array analysis. The cytokines, chemokines and growth factors in the supernatants were detected using a Milliplex Human Cytokine/Chemokine 48-plex kit (Millipore, St. Charles, MO, USA). The cytokine plex-kit includes: sCD40L, EGF, Eotaxin, FGF-2, Flt-3 ligand, Fractalkine, G-CSF, GM-CSF, GROα, IFNα2, IFNγ, IL-1α, IL-1β, IL-1ra, IL-2, IL-3, IL-4, IL-5, IL-6, IL-7, IL-8, IL-9, IL-10, IL-12 (p40), IL-12 (p70), IL-13, IL-15, IL-17A, IL-17E/IL-25, IL-17F, IL-18, IL-22, IL-27, IP-10, MCP-1, MCP-3, M-CSF, MDC (CCL22), MIG, MIP-1α, MIP-1β, PDGF-AA, PDGF-AB/BB, RANTES, TGFα, TNFα, TNFβ and VEGF-A. A multiplexing analysis was performed using the Luminex™ 100 system (Eve Technologies Corp., Calgary, AB, Canada). Each experiment was repeated twice and the means ± SD were calculated. We also used ELISA kits (Cedarlane Canada, Burlington, ON, Canada) to measure FGF-7 levels using the culture supernatants collected 48 h post-ES. Absorbance was measured at 450 nm using a Microplate Reader Model 680 (Bio-Rad, Philadelphia, PA, USA). The sensitivity of the ELISA FGF-7 kit is 15 pg/mL. Four measurements were performed for each condition (*n* = 4).

### 2.10. Cell Behaviors after Exposure to ES and Subculture

To determine whether the effect of ES could be maintained for a longer period following ES, we first exposed the fibroblasts to either 20 or 40 mV/mm for 6 and 24 h and maintained this culture for 48 h without ES. Thereafter, the cells were detached from the PPy/HE/PLLA membranes and washed twice with culture medium, after which 10^5^ cells from each condition were seeded in 6-well plates and cultured for 24, 48 and 72 h. Following each culture period, cell morphology was assessed using an inverted microscope. In another set of experiments performed under the same ES conditions, cell viability was evaluated after 5 days of cell subculture by means of a trypan blue exclusion assay (*n* = 4).

### 2.11. Statistical Analysis

The statistical significance of the differences between the control (non-ES) and the test (with ES) values was determined by One-Way ANOVA. Posteriori comparisons were performed using Tukey’s method. Normality and variance assumptions were verified using the Shapiro–Wilk test and the Brown and Forsythe test, respectively, and all the assumptions were fulfilled. *p* values were declared significant at ≤0.05 and the data were analyzed with GraphPad INSTAT software.

## 3. Results

### 3.1. PPy/HE/PLLA Membrane Characteristics

**Figure 2:**
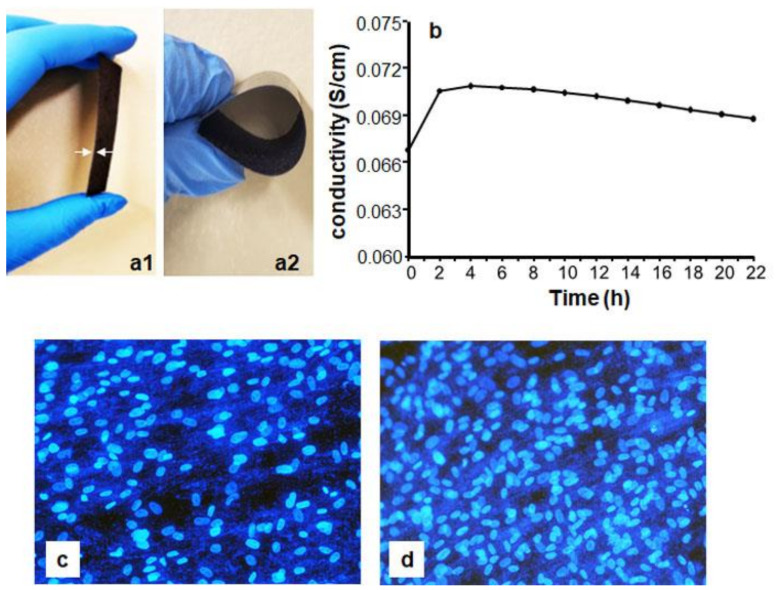
Validation of the conductive PPy/HE/PLLA membrane. Thickness (**a1**), elasticity (**a2**) and electrical conductivity in culture medium (**b**). Membrane biocompatibility with DHSF by cells cultured for 24 h (**c**) and 72 h (**d**) (Hoechst staining).

The thickness of the PPy/HE/PLLA membranes was approximately 0.20–0.34 mm, which provided enough strength and flexibility for handling (Figure 2a). The four-point probe test shows that the PPy/HE/PLLA membranes displayed a surface conductivity of 6.4 × 10^−3^ S/cm (surface resistivity 156.3 Ω·cm). The bulk conductivity of the membranes in culture medium was between 0.06 and 0.07 S/cm (Figure 2b). Following an initial increase caused by water absorption, the conductivity was fairly stable over time. The temperature of the culture medium showed no increase after the current was applied to the membrane (data not shown). Finally, the Hoechst staining results show that the PPy/HE/PLLA membrane was compatible and not toxic to the DHSF, as is evidenced by the apparent good adhesion of the cells on the membrane (Figure 2c,d). These results indicate that the PPy/HE/PLLA membrane was conductive and suitable for DHSF culture.

### 3.2. Optimal ES Parameters for the DHSF 

**Figure 3:**
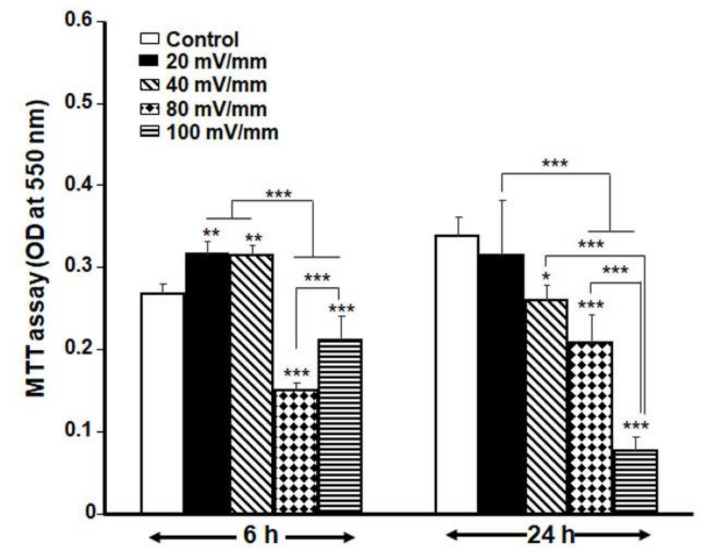
Selection of non-toxic ES intensities for DHSF exposed for 6 or 24 h to ES at various intensities. The cells were cultured for an additional 48 h without ES prior to MTT assay. Data are presented as means ± SD (*n* = 4). * *p* < 0.05, ** *p* < 0.01, *** *p* < 0.001. The control (no ES) and ES-exposed cells were compared, as well as the different ES intensities.

**Figure 4:**
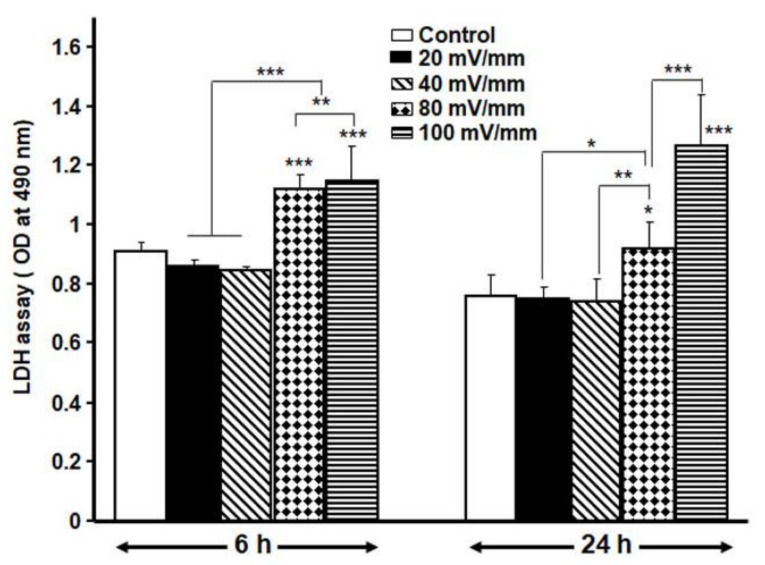
LDH activity of DHSF exposed to different intensities of ES. Data are presented as means ± SD (*n* = 4). * *p* < 0.05, ** *p* < 0.01, *** *p* < 0.001. The control (no ES) and ES-exposed cells were compared, as well as the different ES intensities.

Exposure of DHSF to different intensities and durations of ES reveals that high ES intensities reduced the metabolic activity of the DHSF (Figure 3); indeed, at 100 and 80 mV/mm, the ES decreased the growth/proliferation of the DHSF. However, at low intensities, namely, at 40 and 20 mV/mm and 6 h, fibroblast growth was higher than that observed in the non-exposed cells. In fact, cell proliferation increased significantly (*p* < 0.01), with the absorbance increasing from 0.27 ± 0.01 in the control group to 0.32 ± 0.01 in the 20 mV/mm group and 0.31 ± 0.01 in the 40 mV/mm group (Figure 3). The effect of various intensities of ES on diabetic fibroblast proliferation is confirmed by the LDH activity data. Figure 3 shows high levels of LDH after the cells were exposed to either 100 or 80 mV/mm for both 24 and 6 h, while exposure to 20 or 40 mV/mm showed levels of LDH that were similar to those in the control. Indeed, after exposure of the cells to ES for 6 h, the absorbance increased from 0.91 ± 0.03 in the control to 1.15 ± 0.12 in the 100 mV/mm group and 1.12 ± 0.05 in the 80 mV/mm group. In contrast, the low ES intensities led to non-significant changes in LDH absorbance, which ranged from 0.91 ± 0.03 in the control to 0.85 ± 0.01 in the 40 mV/mm group and 0.86 ± 0.02 in the 20 mV/mm group (Figure 4). The MTT and LDH results thus suggest that ES at 20 mV/mm intensities for 6 and 24 h were the optimal parameters to electrically activate DHSF without causing cell death. Therefore, all subsequent experiments were performed at the 20 and 40 mV/mm intensities. 

### 3.3. Low ES Intensities Promoted DHSF Adhesion, Viability and Growth

**Figure 5:**
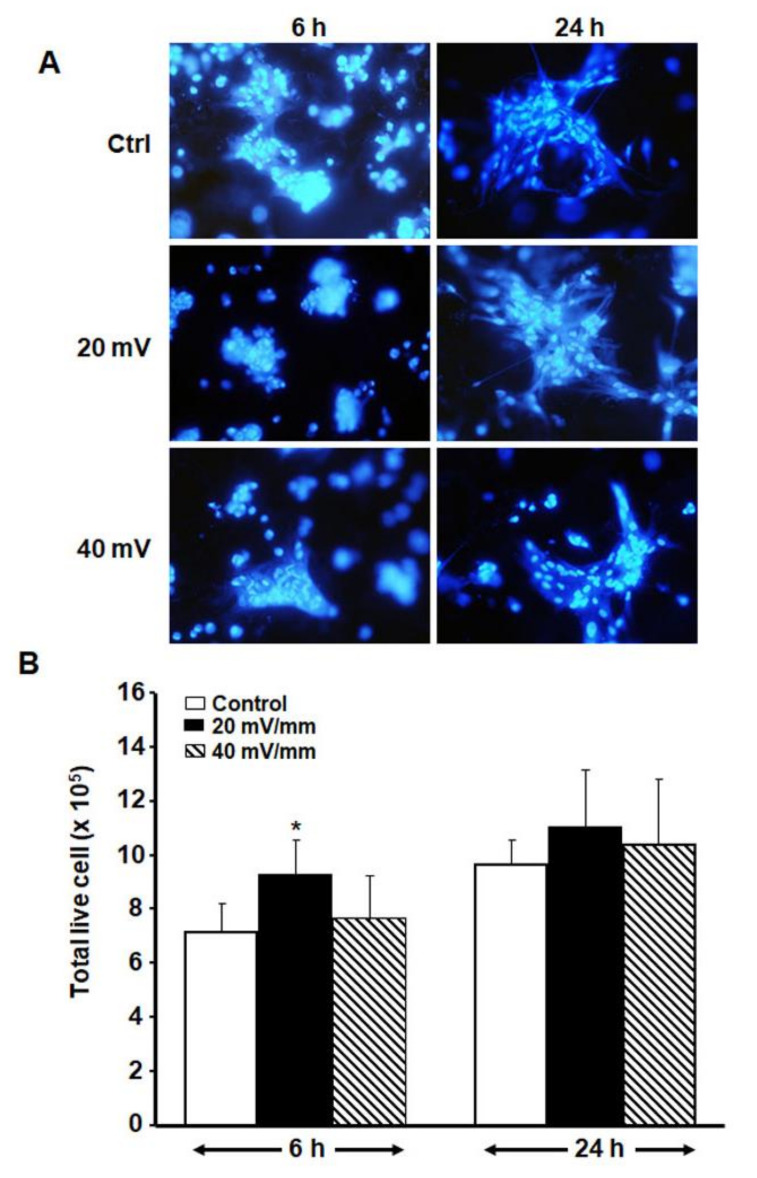
Effect of low-intensity ES on DHSF adhesion and growth. (**A**) Cells on the conductive membrane after 6 or 24 h of ES, with a morphology similar to that in the control. (**B**) Following exposure to ES for 6 or 24 h, then culture for an extra 48 h, the cells were detached from the membranes by means of a 0.05%-trypsin-0.01 EDTA solution and cell suspensions were subjected to a trypan blue exclusion assay to count non-stained (viable) cells using a hemocytometer. * *p* < 0.05. Note the high number of live cells showing the beneficial effect of ES on cell adhesion/growth, basically at 6 h.

Our cell adhesion results show (Figure 5A) that the diabetic fibroblasts adhered over the entire PPy/HE/PLLA membrane, regardless of the presence or absence of ES, while the quantitative analysis results (Figure 5B) reveal that the fibroblasts exposed to ES at 20 and 40 mV/mm maintained a viability comparable to that observed in the control. Of interest is that exposure to 20 mV/mm ES for 6 h significantly increased (*p* < 0.05) the number of live fibroblasts. The 24-h ES also showed a tendency to increase, although not significantly. These results confirm those obtained with MTT and LDH (Figure 3 and Figure 4). 

### 3.4. ES Promoted the Secretion of Various Cytokines and FGF-7 by the DHSF

Table 1 presents the levels of different cytokines measured immediately following ES and 48 h post-ES, showing that several cytokines were either up or downregulated by the ES. Specifically, the level of GM-CSF significantly increased (*p* < 0.01) immediately following the 6-h ES at both 20 and 40 mV/mm. With the 24-h ES, however, we observed a significant (*p* < 0.01) decrease in the level of GM-CSF, which ranged from 162 ± 16 in the control to 118 ± 6 in the 20 mV/mm group and 131 ± 7 in the 40 mV/mm group. This suggests that ES had a biphasic effect on GM-CSF secretion, depending on the exposure time. Further studies are required to investigate this possibility. IL-1β secretion was upregulated in the 6-h stimulation at 40 mV/mm but downregulated in the stimulation at the same intensity but for a longer time (24 h). As for IL-6, its secretion level increased only after the 6-h ES at either 20 or 40 mV/mm, measuring at 892 ± 109 pg/mL in the control, 1187 ± 125 in the 20 mV/mm group (*p* < 0.05) and 1245 ± 171 in the 40 mV/mm group (*p* < 0.01). Of interest is that following exposure to ES for 24 h at both intensity levels, despite an apparent increase in IL-6, this increase was not significant when compared to that in the control. Similar to IL-6, Table 1 also shows the significantly higher IL-8 levels in the ES groups exposed for 6 but not 24 h. Indeed, the level of IL-8 increased from 2104 ± 174 pg/mL in the control to 3893 ± 262 in the 20 mV/mm group (*p* < 0.05) and 5790 ± 853 in the 40 mV/mm group (*p* < 0.01). On the other hand, IL-10 and TNFα secretions were not affected by the ES.

The difference in GM-CSF among groups decreased at 48 h post-ES and mostly ranged between 200 and 240 pg/mL, except for the 6-h ES group at 40 mV/mm, which recorded 309 pg/mL. That said, the two groups stimulated for 6 h once again showed a significant increase compared to that observed in the control (Table 1). IL-1β levels were very stable during the entire culture period (approximately 4 to 5 pg/mL), either immediately after ES or 48 h post-ES. Nevertheless, the two groups stimulated for 24 h did show a statistically significant increase at 48 h post-ES. IL-8 levels increased with the 24-h ES at both 20 and 40 mV/mm, which was not statistically significant, while IL-6 significantly increased after a 24-h exposure to 20 mV/mm. IL-10 decreased following the 6 h exposure to 20 and 40 mV/mm but increased after the 24-h exposure to 20 mV/mm. Finally, TNFα increased 10-fold following exposure to 20 mV/mm for 6 h, compared to that observed in the control, while the 24-h ES had no effect on TNFα secretion by the DHSF (Table 1).

**Figure 6:**
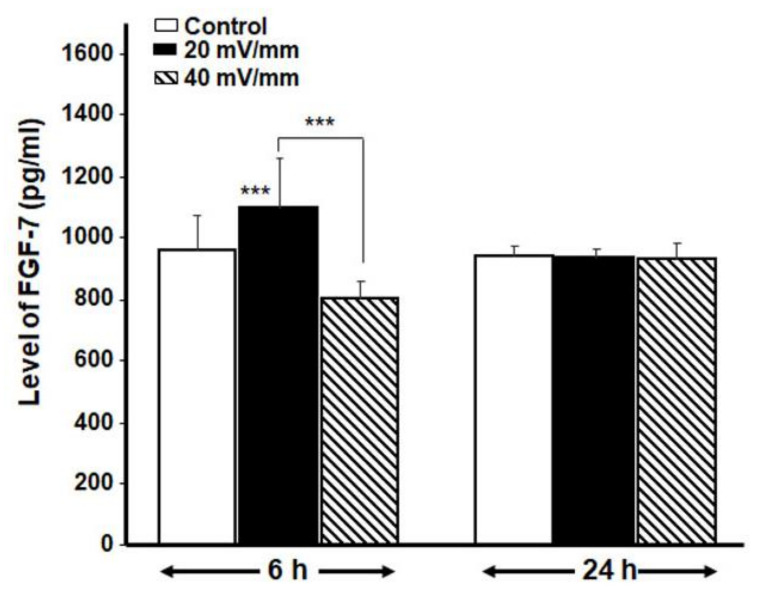
Effect of ES on FGF-7 secretion by the DHSF. ES at 20 mV/mm for 6 h significantly increased FGF-7. Values are means ± SD (*n* = 4). *** *p* < 0.001. The control (no ES) and ES-exposed cells were compared, as well as the different ES intensities.

We also measured FGF-7 by means of ELISA kits. Our data show (Figure 6) a significant (*p* < 0.01) increase in FGF-7 secretion by the fibroblasts stimulated with 20 mV/mm for 6 h. The 24-h ES, on the other hand, had no effect.

### 3.5. Electrically Stimulated DHSF Maintained Their Growth Capacity Even after Subculture

**Figure 7:**
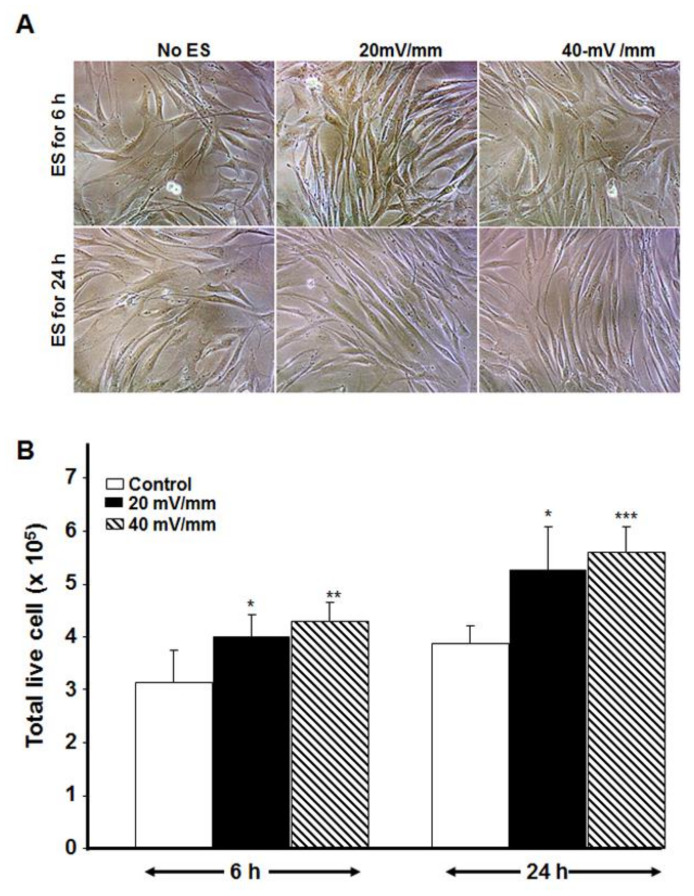
Effect of ES on DHSF subcultures. The ES-stimulated DHSF exhibited the same morphology as that observed in the control (**A**) but proliferated faster than those in the controls (**B**) up to 5 days post-ES. Results are means ± SD (*n* = 4). * *p* < 0.05, ** *p* < 0.01, *** *p* < 0.001.

To ascertain whether the ES-exposed diabetic fibroblasts could maintain their morphology and growth capacity, ES-exposed cells were detached from the PPy/HE/PLLA membranes and subcultured for 5 days in tissue culture dishes. As shown in Figure 7, the morphology of the electrically stimulated cells was similar to that of the fibroblasts in the controls, namely, displaying an elongated cell shape and moderate size, with cytoplasmic condensation and visible nuclei (Figure 7A). The cell count results indicate a significant (*p* < 0.01) increase of viable cells in the ES groups (Figure 7B). It should be noted that with an initial seeding concentration of 10^5^ cells/well, we obtained close to 3.14 × 10^5^ cells in the control, compared to 3.93 × 10^5^ cells in the 20 mV/mm group and 4.31 × 10^5^ cells in the 40 mV/mm group following the 6-h ES. After 24 h of ES, the number of viable cells also increased with both 20 and 40 mV/mm of ES to reach 5.5 × 10^5^ cells. This increase was significant (*p* < 0.05) with the both times and ES intensities. These results confirm that the beneficial effect of ES in promoting DHSF proliferation could be maintained for at least 5 days following electrical stimulation. It should also be noted that with the controls, the 24 h experiments were cultured 18 h longer than were the 6 h experiments, which could explain the different cell numbers recorded.

## 4. Discussion

The delayed healing of diabetic ulcers is a major health concern for millions of patients worldwide. Standard care for these wounds includes debridement, restoration of vascular perfusion, control of infection and the use of appropriate dressings [23]. However, dressings are temporary [24,25], as the best solution is always skin regeneration at the wound site through the growth and migration of the patient’s own cells. For such conditions, the use of autologous skin is the most appropriate treatment [26,27].

One alternative to autologous skin harvesting (which creates a second wound) could be the extraction and use of the diabetic patient’s skin cells, such as fibroblasts; however, diabetic skin fibroblasts are reported to have a slow proliferation rate that limits wound healing. Proliferation of diabetic skin fibroblasts can be improved using non-pharmaceutical stimuli, such as ES.

In this study, we first prepared a conductive, biocompatible PPy/HE/PLLA membrane to show that DHSF could be seeded and maintained in culture on the membrane. These observations concur with previously reported results with fibroblasts extracted from non-diabetic skin donors [19,21]. We then determined the optimal ES intensities contributing to DHSF viability/growth. Here, we demonstrated that the fibroblasts extracted from a diabetic patient were sensitive to high ES intensities. Indeed, only low ES intensities ranging from 20 to 40 mV/mm promoted the proliferation of the DHSF with respect to non-stimulated cells. At high ES intensities (80 or 100 mV/mm) for either a short (6 h) or long (24 h) period, a significant decrease in cell viability was observed, as well as a significant increase in LDH activity, indicating a cytotoxic effect of the strong ES on the DHSF. Of interest is that under the same experimental setup, non-diabetic fibroblasts were found to tolerate ES intensities of up to 200 mV/mm [19,21]. This study therefore reveals, for the first time, that DHSF are more sensitive to ES than are normal fibroblasts, which must be taken into consideration when treating diabetic ulcers.

Since cell adhesion and cell proliferation are two different processes, we performed subsequent experiments with the two lowest ES intensities (20 and 40 mV/mm), showing that both promoted the adhesion of DHSF to the conductive PPy/HE/PLLA membrane. The cells showed a comparable and even higher number at adhesion than what was observed in the control (Figure 5). The cells at adhesion were small and elongated, whether exposed or not to ES, indicating that the low-strength electrical field did not interfere with the cell adhesion process, at least in terms of cell morphology. These are the first observations with DHSF showing that diabetic fibroblasts behave similarly to normal skin fibroblasts when adhering to a substrate in the presence of an electrical current [19,21,28]. The adequate cell adhesion under electrical stimulation thus contributed to better cell proliferation.

We quantitatively evaluated the total live cells following ES exposure using the trypan blue exclusion assay, which revealed the comparable and even higher number of live cells after the exposure to 20 and 40 mV/mm for either 6 or 24 h. This is a very exciting observation, as it points to the possibility of using ES to promote DHSF growth and accelerate wound healing in diabetic patients.

The increased cell proliferation by ES may take place through the increased secretion of cytokines and growth factors. Our cytokine and growth factor analyses confirm an increased secretion of various cytokines, including GM-CSF, IL-1β, IL-6 and IL-8, which all play an active role in the wound healing process [29].

Our findings also show an increased secretion of FGF-7, a member of the fibroblast growth factor family and a major paracrine mediator of epidermal homeostasis through the regulation of keratinocyte proliferation, differentiation and migration [30,31,32]. FGF-7 plays an important physiological role during wound healing [33]. This work therefore demonstrates that appropriate ES may prove useful in promoting the healing of foot ulcers of diabetic patients through the active release of cytokines and growth factors such as FGF-7. Cytokines and growth factors contribute significantly during the wound healing process [34]. Among the principal activated signaling pathways in wound healing are MAPKs [35,36]. MAPK14 was shown to be specifically activated in diabetic patients [37] and could thus be activated by ES, as ES was shown to promote cell proliferation and migration via the activation of MAPKs [38,39].

Since healing takes time, particularly for elderly diabetic patients, we evaluated whether the effect of ES on DHSF could be maintained for a long period of time following exposure to ES. Our results show, for the first time, that subcultured DHSF were able to maintain a significantly higher proliferation rate up to 5 days post-ES, compared to non-stimulated cells (Figure 6). These observations are in agreement with previously reported results with non-diabetic human skin fibroblasts [19,21].

## 5. Conclusions

Diabetic human skin fibroblasts were successfully extracted, cultured and subcultured. The exposure to ES at 20 or 40 mV/mm for 6 and 24 h increased diabetic fibroblast adhesion and proliferation, as well as the secretion of cytokines and FGF-7. This study also demonstrates that the effects of ES on DHSF were maintained for at least 5 days post-exposure to ES. Our overall results therefore suggest that ES could be used to activate diabetic fibroblasts and show potential as a tool to improve the healing of diabetic ulcers.

## Figures and Tables

**Table 1:** Cytokine secretion by diabetic human skin fibroblasts after exposure or not to electrical stimulation. PV = *p*-value; Con. = Concentration; Ns = Not significant; OOR = Out of range.

		6 h			24 h		
		Control	20 mV/mm	40 mV/mm	Control	20 mV/mm	40 mV/mm
**Cytokine levels (pg/mL) measured immediately after stopping ES**							
GM-CSF	Con.	10.8 ± 1.14	16.32 ± 0.59	30.4 ± 2.84	162.0 ± 16.69	118.7 ± 6.47	131.1 ± 7.01
	PV		<0.01	<0.001		<0.001	<0.01
IL-1β	Con.	3.7 ± 0.27	4.3 ± 0.21	4.6 ± 0.46	5.0 ± 0.25	5.0 ± 0.35	4.4 ± 0.12
	PV		Ns	<0.01		Ns	<0.05
IL-6	Con.	892.4 ± 109.9	1187.4 ± 125.4	1245.8 ± 171.9	1285.0 ± 54.6	1459.9 ± 299.6	2189.2 ± 911.5
	PV		<0.05	<0.01		Ns	Ns
IL-8	Con.	2105 ± 175	3894 ± 263	5791 ± 853	8667 ± 2270	9167 ± 376	8253 ± 1069
	PV		<0.05	<0.01		Ns	Ns
IL-10	Con.	OOR	OOR	OOR	0.21 ± 0.14	0.38 ± 0.06	0.11 ± 0.04
	PV		-	-		Ns	Ns
**Cytokine levels (pg/mL) measured 48 h post-exposure to ES**							
GM-CSF	Con.	197.2 ± 17.5	225.6 ± 14.28	309.4 ± 10.62	220.4 ± 27.4	182.6 ± 3.76	240.0 ± 13.6
	PV		<0.05	<0.001		<0.05	Ns
IL-1β	Con.	4.1 ± 0.3	4.4 ± 0.2	4.7 ± 0.5	4.0 ± 0.3	4.6 ± 0.3	4.5 ± 0.2
	PV		Ns	Ns		<0.01	<0.05
IL-6	Con.	2187.6 ± 633	2083.4 ± 690	1414.2 ± 249	1447.7 ± 434	1976.3 ± 293	1555.7 ± 110
	PV		Ns	Ns		<0.05	Ns
IL-8	Con.	17168.7 ± 6959.7	8712.4 ± 1213	8808.7 ± 1080	8580.8 ± 537	9391.1 ± 74	9252.1 ± 1373
	PV		<0.05	<0.05		Ns	Ns
IL-10	Con.	0.8 ± 0.17	0.6 ± 0.08	0.3 ± 0.07	1.0 ± 0.27	1.4 ± 0.16	0.9 ± 0.17
	PV		<0.05	<0.001		<0.05	Ns

## Data Availability

Data is contained within the article.
